# Embryo transfer: past, present, future – a personal perspective

**DOI:** 10.1590/1984-3143-AR2024-0068

**Published:** 2024-08-16

**Authors:** Patrick Lonergan

**Affiliations:** 1 School of Agriculture and Food Science, University College Dublin, Belfield, Dublin 4, Ireland

**Keywords:** assisted reproductive technologies, bovine, IVF

## Abstract

Embryo transfer is just one of a range of assisted reproductive technologies – often the last one in a sequence of others – that has revolutionised the cattle breeding industry. The number of in vitro-produced embryos transferred annually now surpasses the number derived by traditional superovulation by a factor of four. Although issues with cryotolerance of IVP embryos, embryo loss, and, in some cases, calf birth weight remain to be fully resolved, IVP embryos are likely here to stay as a tool for genetic improvement in dairy herds, offering increased flexibility in sire usage allowing multiple pregnancies from elite dam-bull combinations to be generated and the ability to produce more embryos per unit time than traditional superovulation. What follows is a short personal look back at the last 30 years; if you are looking for deep insights into the underlying biology regulating embryo development, this is not the place to look! Please refer to some of the excellent recent reviews and research papers cited herein.

## Introduction

When I received an email from the AETE President Marja Mikkola informing me that I had been selected to receive the 2024 Pioneer Award I have to admit that I was shocked, humbled and a little embarrassed, all at once – shocked, because it was completely unexpected; humbled, because I know the calibre of those who have previously received the award (see Table 1); and embarrassed, not because of false modesty, but because I genuinely consider there are many people more deserving than me. Furthermore, such awards are normally reserved for those near the end of their careers; I hope I still have a few productive years left! Nonetheless, it is a huge honour for me to accept the award and to be listed among those eminent previous recipients. It is a further delight to have my great friend and colleague, Dimitrios Rizos, himself a past President of AETE, present the commendation.

**Table 1:** Past locations of annual meetings of the AETE and past recipients of the AETE Pioneer Award.

**Year**	**Meeting**	**Venue**	**Recipient**
1984-1990	1-6	Lyon, France	-
1991	7	Cambridge, UK	-
1992	8	Lyon, France	-
1993	9	Lyon, France	Joachim Hahn, Germany
1994	10	Lyon, France	Charles Thibault, France
1995	11	Hanover, Germany	Ian Gordon, Ireland
1996	12	Lyon, France	Steen Willadsen, UK
1997	13	Lyon, France	Robert Moor, UK
1998	14	Venice, Italy	Pierre Mauléon, France
1999	15	Lyon, France	Alban Massip, Belgium
2000	16	Santander, Spain	Robert Cassou, France
2001	17	Lyon, France	Josef Fulka, Czech Republic
2002	18	Rolduc, Netherlands	AE ‘Tony’ Wrathall, UK
2003	19	Rostock, Germany	Ian Wilmut, UK
2004	20	Lyons, France	Torben Greve, Denmark
2005	21	Keszthely, Hungary	Jean-Paul Renard, France
2006	22	Zug, Switzerland,	Ray Newcomb, UK
2007	23	Alghero, Sardinia	Steph Dieleman, Netherlands
2008	24	Pau, France	Gottfried Brem, Austria
2009	25	Poznan, Poland	WR ‘Twink’ Allen, UK
2010	26	Kuopio, Finland	Yvan Heyman, France
2011	27	Chester, UK	Maurice Boland, Ireland
2012	28	St Malo, France	Danielle Monniaux, France
2013	29	Istanbul, Turkey	Tom McEvoy, UK
2014	30	Dresden, Germany	Klaus-Peter Brüssow, Germany
2015	31	Ghent, Belgium	Michel Thibier, France
2016	32	Barcelona, Spain	Henrik Callesen, Denmark
2017	33	Bath, UK	Cesare Galli, Italy
2018	34	Nantes, France	Patrice Humblot, France
2019	35	Murcia, Spain	Poul Hyttel, Denmark
2020	36	2020 Online	-
2021	37	2021 Online	-
2022	38	Utrecht, Netherlands	Hilary Dobson, UK
2023	39	Heraklion, Greece	Sabine Meinecke-Tillmann and Burkhard Meinecke, Germany
2024	40	Brescia, Italy	Patrick Lonergan, Ireland

Having to write this short paper has allowed me to pause, ‘take stock’ and look back over my career to date. I remember very well, as a naïve PhD student, attending my first scientific conference (the annual meeting of the International Embryo Technology Society, IETS, in Bournemouth, UK, in January 1991). Not really knowing what to expect, on the first morning, myself and a fellow PhD student from Malaysia, Sharif Haron, walked from our hotel to the conference centre behind two ‘cowboys’ adorned with Stetsons and cowboy boots (pretty sure it was Charles Looney and Brad Stroud from Texas); we wondered what we were letting ourselves in for! I was starstruck seeing some of my ‘heroes’ from the literature for the first time, and realising that they were all (relatively!) normal people. This may be difficult for younger researchers to appreciate given the immediacy of information available online today, where everyone has a web presence in one form or another, but in the pre-internet world, at the risk of sounding old, things were very different.

At that conference, there were talks about alternative gonadotrophins for superovulation in cattle (Maurice Boland, Ireland), a new (!) technique called transvaginal ultrasound guided follicular aspiration of bovine oocytes (Martin Pieterse, The Netherlands), follicular dynamics in sheep and cattle (Marc-Antoine Driancourt, France), turnover of dominant follicles in cattle (Jim Roche, Ireland), oocyte maturation and sperm transport in superovulated cattle (Poul Hyttel, Denmark), cryopreservation of ova and embryos from livestock (Heiner Niemann, Germany), recipient management and embryo transfer (Peter Broadbent, UK), velogenetics for the reduction of generation interval (Michel Georges, Belgium), nuclear transplantation in cattle (Steen Willadsen, Denmark) and a look forward to the next 100 years of embryo transfer (George Seidel, USA). What a stellar line-up of speakers and topics! It was fantastic and I was hooked! As a result, I have only missed two IETS conferences since 1991, have served on the Board of Governors and was lucky enough to be elected President in 2009.

Later the same year, I attended my first AETE conference (the 7^th^ meeting of the association) in Cambridge, UK, in September 1991. This was the first time the meeting was held outside of its birthplace in Lyon, France (Ponsart et al., 2009; Thibier 2014). I drove from Ireland with a fellow PhD student, picking up Dr Ke-Huan Lu en route, a previous student of my PhD mentor, Prof. Ian Gordon, and one of the first to produce a calf from an IVF-derived embryo (Lu et al., 1987, 1988). It was a great meeting and my first exposure to those involved in embryo research in Europe. Back then, Michel Thibier, a central figure in the establishment of the AETE and President that year, would do a simultaneous translation into French of each presentation! There were invited talks on aspects of embryo production in vivo and in vitro (Heiner Niemann, Germany), rapid cryopreservation of bovine embryos (AM de Leeuw, The Netherlands), and extraspecies embryo transfer in equids (WR ‘Twink’ Allen, UK). On the centenary of the first paper to be published on embryo transfer in mammals (Heape, 1891), Chris Polge (UK), who is widely credited with discovering the cryoprotective properties of glycerol (Polge et al, 1949) – although apparently similar work by others predates it (Bernstein and Petropavlovski, 1937; Rostand, 1946) – and who had been instrumental in bringing the meeting to Cambridge, delivered the Walter Heape Memorial Lecture on novel reproductive biotechnologies. In that paper, he described the opportunities for large scale production of embryos in vitro, the potential of sex-sorted semen, embryo multiplication by nuclear transfer and genetic modification to create transgenics. In the three decades since he gave that presentation, many of these technologies have become well established in the tool box of assisted reproductive technologies available to farmers.

I attended most of the AETE meetings in the subsequent years and served on the Board from 2000 to 2007 (Secretary from 2002 to 2007). This was a very enjoyable time working with colleagues from across Europe; as Secretary and the only native English speaker on the Board at the time, I had the pleasure of editing the abstracts each year and through this activity I got to know all of the individuals involved in domestic animal embryo work in Europe. While I have not been able to attend as many meetings as I would have liked in recent years, I have dipped in and out at regular intervals and am happy to see that the AETE continues to be a very friendly group with great science and great social events.

## Early beginnings - my introduction to the world of embryos

I owe a lot to my late uncle, Tom Lonergan, my father’s brother, with whom I spent all of my childhood summers, working on his small dairy farm in County Tipperary in the south of Ireland. This is where my interest in agriculture was born. Mainly because of him, and with my parents’ encouragement, I entered University College Dublin (UCD) as a student in autumn 1984, graduating five years later with a Bachelor’s Degree in Agriculture. At the time, students were obliged to do a full ‘practical year’ (now called professional work experience) to gain experience in all of the main farm enterprises (dairy, beef, sheep, pigs and tillage). I spent much of that year at the University’s Lyons Farm, located some 25 km from the main UCD campus in Dublin, which was later to become my main place of work.

**Figure 1:**
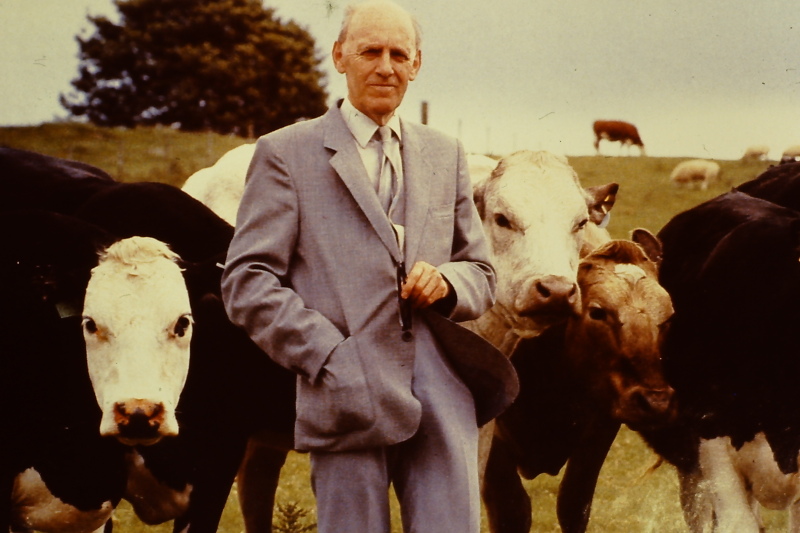
Prof. Ian Gordon (1928-2021) – a true pioneer in the field of livestock reproduction. Despite his international reputation and prolificacy in terms of publications, few published photos exist of him.

I have always loved the challenge of identification. Outside of work, my passion is bird identification and I spend whatever spare time I have either immersed in books on bird identification or in the field watching birds. During the early part of my undergraduate degree in Agricultural Science at UCD, two modules stuck out – one was Agricultural Botany which involved the identification of common grasses and weeds and the other was Plant Pathology which involved, in part, identifying diseases on the leaves of a variety of crop plants. This was done using an identification key and I loved the challenge. I was convinced that I would follow this subject after graduation and become a plant pathologist. Then, in final year, we had Prof. Ian Gordon (Figure 1), himself a past recipient of the AETE Medal (1995) and the IETS Pioneer Award (1998), for the subject of Animal Reproduction. His lectures were an inspiration! One of his greatest characteristics as a teacher was his encyclopaedic knowledge of the literature. As undergraduate students we were riveted by his fascinating lectures, as he regaled us with entertaining anecdotes and stories from his own experience and from the literature (collecting urine from nuns in Italian convents to purify human menopausal gonadotropin was particularly memorable!). My ‘passion’ for plant pathology was quickly forgotten and I decided that further study in mammalian reproduction was for me.

I started my venture into the world of bovine embryology in 1989 when I began a Masters under the supervision of Prof Gordon, or ‘Prof’ as he was affectionately known by all of his graduate students. Gordon graduated from Nottingham University in 1951 before embarking on a prolific scientific career under the guidance of Sir John Hammond at Cambridge, widely regarded as the father of modern animal physiology. Gordon was a true pioneer in the field of livestock reproduction, particularly in the area of oestrous synchronisation, superovulation and non-surgical embryo transfer and, latterly, in the area of in vitro embryo production in cattle. He was a prolific author, publishing many scientific articles with some 280 to his credit, but in his later years at UCD, from where he retired in 1993, and in his retirement, he published 6 books. His first book, *Controlled Breeding in Farm Animals,* was published in 1983. This later evolved into a four-volume series entitled *Controlled Reproduction in Farm Animals* separately dealing with Cattle and Buffalos (1996), Sheep and Goats (1997), Horse, Deer and Camelids (1997) and Pigs (1999). The second edition of his book *Reproductive Technologies in Farm Animals*, first published in 2004, was published as recently as 2017. In addition, his masterpiece, *Laboratory Production of Cattle Embryos* was first published in 1994 with a second edition published in 2003. This tome was a true ‘one-stop-shop’ covering everything from the historical developments in IVF technology in cattle, through detailed chapters on oocyte recovery, oocyte maturation, sperm capacitation, IVF, embryo culture, cryopreservation, to embryo transfer. Although, a little dated given the rapid progress over the past 20 years, this book is still a ‘must have’ for anyone working in the area. And remember, all of this was written by painstakingly manually wading through hard copies of published journals and books of abstracts, without the aid of a ‘Google Search’ or ‘PubMed’ which we all rely on so much today. Ian Gordon died in 2021; indeed that was a bad year for domestic animal reproductive biologists – as well as Gordon, we lost George Seidel (Colorado State University), Twink Allen (University of Cambridge) and Keith Inskeep (West Virginia University), all pioneers in their own right.

During my Masters, I was based in what became known as the ‘IVF Lab’ at UCD’s Lyons Research Farm. Little did I know then that I would, after a few years as a post-doc abroad, spend my entire career at Lyons. We were (and still are) lucky to be located very close to several major abattoirs from which, through their continued support, we have unlimited access to ovaries for research. During my Masters, I investigated various factors affecting the production of embryos in vitro including the effect of breed cross of donor, stage of the oestrous cycle and donor age on oocyte yield, the effect of follicle size on oocyte diameter and meiotic competence, the effect of temperature (10 v 30 degrees) and duration of storage (up to 24 h) of ovary collection on oocyte quality and the optimum heparin concentration for IVF.

After my Masters, I continued in the area of embryo production in vitro and completed a PhD, also under Prof Gordon’s supervision, investigating various factors affecting in vitro embryo production including the effect of cumulus-oocyte-complex morphology, follicle size, follicular fluid supplementation and alternatives to serum during IVM on the outcome of IVP. In addition, we investigated the effect of priming the ovary with FSH prior to ovary collection on blastocyst yield after IVF. My first paper (and second most cited) was published in 1994 on the effect of follicle size on bovine oocyte quality and developmental competence following maturation, fertilization, and culture in vitro (Lonergan et al., 1994). Looking back, it was a very simple study – very different from what is expected by journals nowadays.

It was at Lyons that I met and fell in love with Trudee – while I was finishing my PhD, she completed a Masters. We were very fortunate to be among Gordon’s last cohort of graduate students. Amongst the meetings we attended together was the 12^th^ International Congress on Animal Reproduction (ICAR) in The Hague in August 1992 – another feast of excellent science. Amongst the line-up of speakers, a young Poul Hyttel (AETE Pioneer in 2019) from the then Royal Veterinary and Agricultural University (KVL; now, the University of Copenhagen) gave a fantastic workshop on oocyte growth and development including beautiful electron microscopy images of oocytes during maturation in vivo and in vitro. Trudee decided almost then and there that she was going to do a PhD with Poul and in 1993 she was awarded one of the first EU Marie Curie International Fellowships to do just that (in collaboration with Torben Greve, AETE Pioneer in 2004). So began a lifelong friendship with Poul and Trudee’s research career in oocyte biology.

In November 1992, after completing my PhD, I took up a one-year post-doctoral position at the Norwegian Veterinary School in Oslo working with Wenche Farstad. That was a fantastic year and gave me the opportunity to get to know all of Scandinavia; leaving Oslo at 4 am to drive to the nearest abattoir, in Hamar, was a bit of a shock to the system, as was getting stopped by the police for speeding at 6 am ‘in the middle of nowhere’ and receiving a very hefty fine (honestly officer, I wasn’t going that fast)! I subsequently moved to the Institut National de la Recherche Agronomique (INRA) in Nouzilly near Tours in the Loire Valley, France, where I spent the next four years working with Pascal Mermillod on various aspects of oocyte and embryo development in vitro. INRA was a great place to work. Once I ‘mastered’ French, with the persistence and indulgence of people like Pascal, Nati Poulin (‘Eh ben dis donc!’) and others, I integrated completely. At the time, as is still the case today, INRA-PRMD (Physiologie de Reproduction des Mammifères Domestiques) was a melting pot of leading experts and their teams working on all aspects of mammalian reproduction including the recently retired Pierre Mauléon (first President of AETE), Marc-Antoine Driancourt (follicle development), Danielle Monniaux (follicle growth), Pascal Mermillod (oocyte maturation, bovine IVF), Yves Cognie and Gerard Baril (small ruminant reproduction), Eric Palmer (equine reproduction), Yves Combarnous (Molecular Endocrinology), Michel Terqui, Francoise Martinat-Botté and Francoise Berthelot (pig reproduction), Jean-Louis Dacheux (epididymis function), Michel Courot (andrology and male fertility), Jean-Pierre Signoret (animal behaviour), Philippe Chemineau (photoperiod), Marie-Thérèse Hochereau-de Reviers (spermatogonial stem cells) and many others. In addition, through involvement in a European FP3 Consortium grant (‘Ex Ovo Omnia’) during my time at INRA, coordinated by Franz Dessy at Louvain Le-Neuve in Belgium, I got to know many new colleagues from across Europe.

While in France, in 1994, KVL offered an oocyte/embryo-focused summer school in Copenhagen. This was a fully funded week-long series of lectures and practicals with many of the leading figures in oocyte and embryo biology at the time (including, from memory, Ian Wilmut, Jan Motlik, Taku Nagai, Barry Bavister, Heiner Niemann, Don Rieger, Frank Barnes, and many others). We had lectures by day and socialised at night in a very informal atmosphere that allowed students to rub shoulders with some of the ‘greats’ of the day; it was wonderful.

Trudee finished her PhD in Copenhagen in 1996 and came to France for the last year or so; during that time we got married and had our first child, Tadhg, who was born in Tours. We returned to Ireland in the summer of 1997 and that autumn I returned to UCD as a Post-doc working with Maurice Boland (also a recipient of the AETE Pioneer Award, in 2011) and was appointed to the faculty in the Department of Animal Science and Production in September 2001, where I have happily remained to this day.

## Importance of collaboration

Collaboration has played a major role in my career. I have been lucky to establish a large network of excellent colleagues at home and internationally through national and international grants as well as through one-to-one contacts. This has resulted in the publication of many papers together and the co-supervision of a large number of graduate students. Apart from Trudee, with whom, at the time of writing, I have co-authored 72 papers, Dimitrios and I have published 74 papers together since our first in 1994 when he was a visiting Erasmus student and then from his PhD at UCD before he moved to INIA-Madrid in 2004. Other notable collaborators include Sean Fair, University of Limerick (51 papers), Alfonso Gutiérrez-Adán, INIA-Madrid (44 papers), Stephen Butler, Teagasc (42 papers), David Kenny, Teagasc (33 papers), and Tom Spencer, University of Missouri (28 papers). There are many others – too many to mention – but of course, most of these publications arose from the hard work of a team of excellent graduate students (Table 2).

**Table 2:** List of graduate students (co-)supervised.

**Student**	**Year**	**Masters/PhD**	**Thesis title**
Martina O’Kearney Flynn	1998	Masters	Culture of bovine embryos in vitro
Garret Byrne	1999	Masters	Effect of freezing rate of ram spermatozoa on subsequent fertility in vivo and in vitro
Brian Enright	1999	Masters	Culture of in vitro produced bovine zygotes in vitro vs in vivo: implications for early embryo development and quality
Michael O’Leary	2001	Masters	Effect of Organic and Inorganic Selenium Supplementation on aspects of Reproduction and Tissue Concentration
Mark Kingston	2003	Masters	Factors Affecting Embryo Production in Cattle
Wendy Griffin	2004	Masters	The Effect of Dosing Propylene Glycol to Dairy Cows During the Early Postpartum Period, or to Heifers on Metabolic and Developmental Parameters Related to Fertility
Lisa Burke	2004	Masters	The Developmental Competence of Oocytes in Dairy Heifers and Cows
Catherine Foley	2005	Masters	In vitro production of bovine embryos from single oocytes
Adam Woods	2006	Masters	The Effect of Embryo Source and Recipient Progesterone Environment on Embryo Development in Cattle
Catherine Lawson	2008	Masters	The effect of omega-3 polyunsaturated fatty acids on early embryo development in cattle
Miriam de Feu	2008	Masters	The Effect of Genotype And Management Factors on Fertility And Nutrition in The Modern Holstein - Friesian Dairy Cow
Lydia O’Hara	2009	Masters	Effect of storage duration, storage temperature and diluent on the viability and fertility of fresh ram sperm
Michael McDonald	2014	Masters	The relationship between ear temperature and onset of oestrus and ovulation in beef heifers
Nicola Gillespie	2014	Masters	Taught Masters in Animal Reproduction
Christopher Johnston	2014	Masters	Taught Masters in Animal Reproduction
John Doyle	2014	Masters	Taught Masters in Animal Reproduction
Niamh Cantwell	2019	Masters	Studies affecting in vitro development of bovine oocytes and embryos
Evelyn Drake	2020	Masters	Evaluation of delayed timing of artificial insemination with sex-sorted spermatozoa on pregnancy/artificial insemination in seasonal calving, pasture-based, lactating dairy cows
Rachel White	2022	Masters	Incidence and treatment of endometritis and effect of dietary marine seaweed extracts on reproductive function in spring-calving, pasture-based lactating dairy cows
Jane Kennedy	2023	Masters	Factors affecting in vitro production of bovine embryos
Fabian Ward	2002	PhD	The Use of Ovum Pick-Up in Association with In Vitro Maturation, Fertilisation and Culture as an Aid to Improved Reproduction in Cattle
Dimitrios Rizos	2002	PhD	Studies on Development, Cryotolerance, Ultrastructural Morphology and Gene Expression in Bovine Embryos Produced In Vivo or In Vitro
Serafeim Papadopoulos	2003	PhD	Studies in the Production of Ruminant Embryos
Sean Fair	2005	PhD	Ewe breed difference in fertility after AI with frozen semen
Ciara O’Meara	2005	PhD	Evaluation of semen for artificial insemination of sheep
Deirdre Corcoran	2006	PhD	Gene expression during development in bovine embryos
Niamh Forde	2007	PhD	Genomics of ovarian follicle development
Anna Zielak	2007	PhD	Identification of novel genes regulating ovarian follicle development
Fiona Carter	2009	PhD	Gene expression during bovine oocyte maturation and early embryo development
Naomi Smith	2009	PhD	Impact of maternal nutrition during gestation on offspring health and development
Lorraine Richardson	2011	PhD	Cervical function: explaining fertility differences among breeds of ewe
Sean Cummins	2012	PhD	The effects of predicted differences for fertility in dairy cows on productive efficiency and gene expression profiles in key tissues
Ian Hutchinson	2012	PhD	The effect of strategic supplementation with polyunsaturated fatty acids on the reproductive performance of lactating dairy cattle
Abdullah Al Naib	2013	PhD	In vitro embryo production in cattle: a tool to understand the regulation of sperm function and embryo development
Lydia O’Hara	2014	PhD	Progesterone regulation of conceptus development in cattle
Lilian Okumu	2010	PhD	Localisation of key genes in the bovine uterus during the oestrous cycle and early pregnancy
Satoko Matoba	2013	PhD	Studies of oocyte developmental competence in cattle
Beatriz Fernández-Fuertes	2016	PhD	Factors affecting sperm function in cattle
Shane Leane	2016	PhD	Nutritional effects on reproduction in pasture-based systems of dairy production
Francis Curran	2016	PhD	Nutritional effects on fertility in pasture-based systems
Federico Randi	2017	PhD	An integrated approach to improving the reproductive efficiency of season-calving cow herds in Ireland
Colin Byrne	2017	PhD	An examination of the effects of nutrition on age at puberty and subsequent fertility inn dairy-bred bulls
Edel Murphy	2018	PhD	Optimising semen processing procedures of liquid and frozen-thawed bull semen in a commercial artificial insemination centre
Claudia Passaro	2018	PhD	Embryo-endometrial interaction during early pregnancy in cattle
José María Sánchez Gómez	2018	PhD	Understanding conceptus-maternal interaction in cattle to improve embryo survival
Eber Rojas Cañadas	2018	PhD	Fertility phenotypes in season-calving pasture-based dairy cows
Clio Maicas	2019	PhD	Fertility of sex-sorted sperm in seasonal-calving pasture-based dairy herds
Beatriz Rodriguez Alonso	2019	PhD	Studies on embryo-maternal interaction in the oviduct of cattle
Benjamin Planells Codoner	2019	PhD	New molecular insights into sex determination and early differentiation in mice and cattle
Sandra Bagés Arnal	2020	PhD	Studies on maternal embryo communication in cattle
Stephen Coen	2022	PhD	Nutrition and genomic control of sexual maturation in the bull
Elena O’Callaghan	2022	PhD	Sire contribution to pregnancy establishment in cattle
Alan Crowe	2024	PhD	Use of assisted reproduction techniques to accelerate genetic gain and increase value of beef production in dairy herds
Laura Thompson	In progress	PhD	Effect of assisted reproduction techniques on foetal development and postnatal characteristics of calves
Eliza O’Shea Murphy	In progress	PhD	Accelerating genetic gain and improving beef output from dairy herds
Joanne Hanifin	In progress	PhD	Effect of heterospermic semen on fertility in cattle

## Progesterone, the uterus and conceptus elongation

In vitro embryo production is a fascinating tool. It is possible to replicate in a petri dish the final stages of oocyte maturation in the follicle, fertilisation in the oviduct and the first week or so of embryo development in the oviduct and uterus. Thus, the early embryo is somewhat autonomous; it does not require contact with the female reproductive tract to reach the blastocyst stage and is capable of establishing a pregnancy after transfer to a uterus that itself has not been exposed to an embryo prior to the transfer. Having spent a lot of my early career trying to optimise the in vitro production of embryos, in the early 2000s I became more interested in maternal embryo communication; growing embryos in the lab was one thing but how they interact with the female reproductive tract to establish a pregnancy was much more interesting. We first used a combination of superovulation, artificial insemination and in vivo vulture in the sheep oviduct to tease out the respective impacts of oocyte maturation, fertilisation and embryo culture in vivo vs. in vitro on embryo yield and embryo quality. These data were published in one of my favourite, and our most cited, paper (Rizos et al., 2002) and were most recently kindly highlighted by Pete Hansen in his paper associated with the 11th International Ruminant Reproduction Symposium in Galway, Ireland in May 2023 (Hansen, 2023). The broad conclusion from these studies, was that the main factor determining blastocyst yield in vitro is the quality of the oocyte that goes into maturation while the main factor affecting blastocyst quality is the post-fertilisation culture environment. To a large degree, the developmental competence of the oocyte is ‘set’ once it is removed from the follicle and few, if any, protocols for in vitro maturation result in a consistent improvement in development above the typical 30-40% blastocyst rate (Lonergan and Fair 2016). These results were subsequently extended by us (Lonergan et al., 2003a,b) and others (Gad et al., 2012) by carrying out reciprocal transfers between in vitro and in vivo culture conditions.

We carried out a series of studies in collaboration with Urban Besenfelder and Viteslav Havlicek from the University of Vienna, using Urban’s exquisite endoscopic transfer method, to place embryos (up to 50) into the oviducts of females in different metabolic states (nulliparous heifers, lactating and nonlactating postpartum cows) and were able to demonstrate that the ability of the oviduct to support early embryo development was compromised due to the metabolic stress associated with lactation (Rizos et al., 2010; Maillo et al., 2012, 2015). More recently, we showed that this is likely, at least partly, due to altered embryonic genome activation (Rabaglino et al., 2023). We also used the same model to understand asynchrony in the oviduct (Rodríguez-Alonso et al., 2020) and the effect of progesterone concentration on development to the blastocyst stage in vivo (Carter et al., 2010).

Together with Jim Roche, Niamh Forde and others we looked at the influence of circulating progesterone on various aspects of uterine biology and embryo development. The first of many papers from those studies described the endometrial transcriptome in heifers during the oestrous cycle and early pregnancy and how this is influenced by increasing progesterone concentrations after ovulation (Forde et al., 2009). This study led to many others through which we attempted to untangle the complex interplay between progesterone, uterine biology and pre-attachment conceptus development (Carter et al., 2010; Forde et al., 2010, 2011a,b, 2012 and others). For more details, see reviews by Spencer et al. (2016), Lonergan et al. (2016), and Lonergan and Sánchez (2020).

Progesterone priming of the uterus is essential for optimal pregnancy establishment. As the corpus luteum develops following ovulation, the uterus is exposed to increasing concentrations of progesterone which alter the transcriptome of the endometrium. By comparing the transcriptome of cyclic and pregnant bovine endometrium, it is clear that temporal changes in endometrial gene expression occur irrespective of whether the cow is pregnant or not and it is really only at the time of maternal recognition of pregnancy at around d 16 that major changes in gene expression are detectable between pregnancy and cyclic animals (Forde et al., 2011a). An adequate rise in progesterone after ovulation drives these normal temporal changes that occur in the endometrial transcriptome of cattle that are necessary for the establishment of uterine receptivity and the promotion of conceptus development. Forde et al. (2009) described the global transcriptome of the endometrium from Day 5 to Day 16 in pregnant and cyclic cattle under conditions of normal and elevated progesterone and revealed how circulating concentrations of progesterone regulate endometrial genes. Those studies found that progesterone supplementation advances the normal temporal changes in endometrial gene expression, particularly for genes associated with energy sources or contributors to histotroph, which may contribute to advanced conceptus development on Day 13 and Day 16. In contrast, low progesterone was associated with an altered endometrial transcriptome and retarded conceptus elongation (Forde et al., 2011b, 2012). Interestingly, the embryo does not have to be present in the uterus during the period of progesterone elevation in order to benefit from it (Clemente et al., 2009), supporting the concept that the positive effect on conceptus growth is mediated via progesterone-induced changes in the endometrial transcriptome.

Around the same time, we carried out one of the first RNA Seq studies on bovine embryos to describe the transcriptomic landscape of the developing embryo from the blastocyst stage on Day 7 to the elongated conceptus on Day 16 (Mamo et al., 2011) and combined these data with similar data from the endometrium to provide a comprehensive list of potentially secreted molecules in the conceptus that interact with receptors on the endometrium and vice versa during the critical window of maternal recognition of pregnancy (Mamo et al., 2012).

We investigated conceptus-induced changes in the endometrial transcriptome to address the question of how soon the cow knows she is pregnant (Forde et al., 2011a). By comparing endometria from cyclic or pregnant heifers on Days 5, 7, 13 and 16, the earliest we could detect differences between cyclic and pregnant animals was on Day 16, by which time most of the changes are due to conceptus-derived interferon-tau. Interestingly, interferon-tau (IFNT) mRNA is detectable in the bovine embryo from day 6 (late morula/early blastocyst stage) onwards (Wrenzycki et al., 1999). Furthermore, bovine blastocysts secrete IFNT into culture medium in vitro (Larson et al., 2001; Kubisch et al., 2004). Culture of bovine endometrial explants in vitro with Day 8 blastocysts leads to an increase in the transcript abundance of several interferon-stimulated genes (ISGs; e.g., *ISG15, MX2* etc), demonstrating that the endometrium can respond to blastocyst-derived IFNT. In contrast, exposure of explants to oocytes, 2-cell embryos or Day 5 morulae did not alter their relative abundance (Passaro et al., 2018, 2019). While some authors, including us, failed to detect a response of the endometrium to pregnancy before approximately Day 15/16 (e.g., Forde et al., 2009), others have reported altered expression of several genes including ISGs by Day 7 (Sponchiado et al., 2017). Thus, there is compelling evidence that the blastocyst as early as Day 7 produces IFNT and that this induces a response in the endometrium. However, whether this interaction has any significant role in pregnancy establishment is probably questionable given that it is possible to transfer embryos into a uterus up to about Day 16 and establish a pregnancy (Betteridge et al., 1980). Indeed, we have shown that the effect of IFNT on the endometrium is very acute, with exposure for as little as 3 h in vitro increasing mRNA expression of a range of ISGs (Talukder et al., 2023).

## Conceptus elongation

The relationship between circulating progesterone and uterine receptivity has been well described (reviewed by Spencer et al., 2016; Lonergan and Sanchez, 2020). Elevated progesterone concentrations in the first week after conception have been associated with accelerated post-hatching conceptus elongation, mediated through advancement in the regular temporal changes in the uterine endometrial transcriptome (Forde et al., 2009) and alterations in the uterine lumen fluid (ULF) composition (Simintiras et al., 2019a).

As stated above, the success of in vitro fertilization (IVF), where embryos are made in the laboratory, demonstrates that contact with the female reproductive tract is not necessary in order for the embryo to reach the hatched blastocyst stage. However, the characteristic elongation of the ruminant conceptus prior to implantation is dependent on secretions from the uterus as evidenced by the fact that it does not occur in vitro and does not occur in vivo in the absence of uterine glands (Gray et al., 2002). This highlights the key role played by the uterine endometrium in driving the elongation process via endometrial secretions which compose the uterine lumen fluid. Temporal changes of the endometrial transcriptome and uterine fluid composition are necessary to establish uterine receptivity to implantation and, in turn, are pivotal to the success of pregnancy establishment. These modifications are regulated by conceptus-derived IFNT together with maternally-derived progesterone from the corpus luteum, to induce expression of genes in uterine luminal and superficial glandular epithelia for transport and/or secretion into the uterine lumen to support growth and development of the conceptus (Simintiras et al., 2019a,b,c). Interestingly, elongation also appears to be associated with oocyte quality as we have observed over numerous studies that IVP blastocysts transferred in groups to the same uterus elongate at different rates. This is important because short (retarded) conceptuses have a different gene expression pattern to their longer age-matched counterparts (Barnwell et al., 2016, Ribeiro et al., 2016) and such short conceptuses produce less IFNT and fail to elicit an appropriate response from the endometrium around the time of pregnancy recognition (Sánchez et al., 2019).

## Beef on dairy

Despite working with embryos and IVF for all of my career, it is only relatively recently that we have had the opportunity to carry out some large-scale embryo transfer studies at a commercial scale (Crowe et al., 2024b). These studies have been carried out in an era when the use of both sex-sorted dairy semen (to generate replacement females) and conventional beef semen (to generate all remaining pregnancies) is increasing in the dairy herd, facilitating genetic gain in replacement females while enhancing the beef value of surplus calves (reviewed by Crowe et al., 2021). Although beef-cross calves have greater economic value than male dairy calves, further gains are potentially feasible through the transfer of purebred beef embryos.

Since 2017, the number of in vitro-produced (IVP) embryos transferred has surpassed the number derived by traditional superovulation, now accounting for approximately 80% of all bovine embryos produced and transferred (Viana, 2023). According to the latest data available from the International Embryo Technology Society, almost 1.2 million IVP embryos were transferred worldwide in 2022 compared to 370,000 in vivo derived embryos (Viana, 2023). Although issues with cryotolerance (i.e., freezability) of IVP embryos, embryo loss, and, in some cases, calf birth weight remain to be fully resolved, IVP embryos are likely here to stay as a tool for genetic improvement in dairy herds, offering increased flexibility in sire usage allowing multiple pregnancies from elite dam-bull combinations to be generated and the ability to produce more embryos per unit time than traditional superovulation.

To test the feasibility of using IVF in our seasonal pasture-based system of production in Ireland, we recently carried out a large-scale field trial to examine fertility in lactating dairy cows following timed AI or timed ET with fresh or frozen, beef or dairy, IVP embryos (Crowe et al., 2024b). Pregnancy rates for embryos transferred fresh were comparable with those achieved after AI. However, consistent with other studies, embryonic loss was increased with IVP embryos compared to AI. A subsequent study (Crowe et al., Forthcoming) examining the timing and incidence of pregnancy loss in the same cohort of cows from service event to parturition revealed that the largest proportion of pregnancy loss occurred before Day 18 (AI and ET). Pregnancy loss from Day 32 to Day 62 was greater following ET compared with AI, particularly with frozen embryos while losses after Day 62 were small (≤ 3.5%) in all groups. The percentage of cows that calved following fresh ET was similar to AI (both greater than frozen ET). Further work is clearly required to improve the likelihood of pregnancy establishment and reduce embryonic and fetal mortality following transfer of a cryopreserved IVP embryo. It is likely, based in recent studies, that some of this loss is due to delayed attachment in IVP embryos (Crowe et al., 2024a)

Pregnancy loss in dairy cows is a major contributor to reproductive inefficiency at herd level (Wiltbank et al., 2016; Berg et al., 2022). Greater embryo mortality presents an obstacle to more widespread use of IVP embryos, particularly in seasonal systems of production with a compact breeding season. This is particularly true in seasonal, pasture-based systems of production with a short, well-defined, breeding season such as that operated in Ireland. In his paper, written as entertainment during the Covid-19 pandemic, Pete Hansen (Hansen, 2020) addressed the incompletely fulfilled promise of embryo transfer in cattle, asking why pregnancy rates are not greater than they are given that embryo transfer bypasses any potential issues relating to oocyte quality, fertilisation and oviduct function. Nonetheless, pregnancy success is generally similar for ET and AI. This would suggest that issues around embryo quality and/or technical improvements in the methodology of ET and recipient management still remain.

## Conclusion

The world of animal reproductive biology is a small one; most of us know, or know of, each other (and most of us review each other’s papers!). I consider myself very lucky to have the job I have; I look forward to going to work every day. Through science, we have been able to visit virtually every corner of the globe and spend time with colleagues, many of whom have become close over the years. There are not a lot of jobs that offer such perks. Our knowledge of reproductive biology and the regulation of early embryo development in domestic animals has come on in leaps and bounds over the past three decades. However, there is still much to discover. My brother, Gerard, often teases me by asking why we have not yet solved the ‘problem’ of cow fertility. We’re too clever I tell him; sure, that would be like turkeys voting for Christmas!
